# Intraobserver reliability and validity of a single ultrasonic measurement of the lateral condyle-capsule distance in the temporomandibular joint

**DOI:** 10.1007/s40477-023-00818-z

**Published:** 2023-09-01

**Authors:** Raquel Delgado-Delgado, Orlando Conde-Vázquez, Fiona Mc Fall, Tomás Fernández-Rodríguez

**Affiliations:** 1 Faculty of Health Sciences, Physical Therapy Department, University of Camilo José Cela, Madrid, Spain; 2 Departamento de Bioloxía Funcional e Ciencias da Saúde, Universidade de Vigo, 36005 Pontevedra, Spain

**Keywords:** Temporomandibular joint, Confidence intervals, Reproducibility of results, Adult, Humans

## Abstract

**Purpose:**

The purpose of the study was to examine the reliability and validity of a single lateral condyle-capsule distance (LCCD) measurement while saving on economic costs and clinical resources.

**Methods:**

A longitudinal test–retest design was used to assess the reliability and validity of single-examiner measures over 72 TMJ sonographic analyses. Intraclass correlation coefficients (ICC) and a Bland–Altman plot were used to study reliability and validity, comparing the first measurement of the LCCD to the mean of 3 measurements taken one week later by the same examiner.

**Results:**

ICC show intraobserver reliability of 0.981, 95% confidence intervals (CI) of 0.969 to 0.988. The mean difference between the ultrasound measurements is 0.019 mm (95% CI 0.0005–0.0383) with a standard deviation of 0.080 mm, demonstrating robust validity. The 95% Limits of Agreement (LoA) are − 0.138 for the lower limit and 0.177 for the upper. Mean relative error is 0.009 mm.

**Conclusion:**

Intraobserver reliability of a trained examiner is very high in the single measurement of the LCCD and validity is significant compared to more complex methods. The risk of bias is low since the mean of three LCCD measurements is calculated as opposed to recording only one single measurement.

## Introduction

Temporomandibular disorders (TMD) are defined as a group of painful conditions affecting the bony structure and soft tissue of the orofacial region. The most common signs and symptoms include separate muscle or joint pain (or a combination of both), limitations to jaw opening, temporomandibular joint noises (e.g., clicking, crepitus, grating, popping), and headache [1–3].

TMD is a serious public health problem affecting 5–12% of the population. It is the second most common musculoskeletal condition after chronic low back pain with a high economic impact—up to $4 billion in the USA [4]. Cross-sectional studies show that women are 1.5–2 times more likely to suffer from TMD than men. The symptoms of TMD in women are presented as more frequent and more severe than those of men [5].

TMD includes intra-articular (e.g., congenital, or developmental disorders, disc derangement disorders, degenerative joint disorders, infection, neoplasia, hypomobility, hypermobility, and trauma) and extra-articular disorders (myalgia, local myalgia, myofascial pain syndromes, myositis, and myospasm) caused by multiple biologic, environmental, social, and psychological factors [2, 6].

The diagnosis of TMD is based on both clinical and physical examination supported by specialized radiological imaging [4, 7, 8]. Currently, magnetic resonance imaging (MRI) is considered the gold standard for the evaluation of the temporomandibular joint and its adjacent structures. This diagnostic method provides detailed information about the position and morphology of the articular disc, the mandibular bone structure, the adjacent soft tissues, as well as possible joint effusions [9–12]. It also allows for dynamic assessment of the translational movement of the mandible. There are, however, limitations to this method, such as low availability and accessibility. The imaging process takes a long time, is costly and may cause discomfort to the patient [12, 13].

In the last twenty years, ultrasonography (US) has been used as a new method for diagnosing TMD. It has the advantage of being non-invasive and less expensive than any other previously used technique. However, some authors found it difficult to observe the articular disc in their examinations and tried to describe indirect echographic signs to determine the disc position. Certain researchers described the distance between the most lateral point of the mandibular condyle and the most lateral point of the articular capsule: the lateral condyle-capsule distance (LCCD) as a possible objective estimation of the disc position [14–16]. Hayashi et al. [16] determined the LCCD in 18 Japanese children and compared their results against magnetic resonance imaging (MR) and helical computerized tomography scans (CT), showing a sensitivity of 83%, a specificity of 96%, and an accuracy of 92% for identifying disc displacement when the LCCD was ≥ 4 mm. This measurement was recommended when assessing lateral disc displacement as it shows good intra-observer reliability (ICC = 0.83) measured in the longitudinal axis and in a closed-mouth position [15]. Recent research supports the use of LCCD on the transverse plane and in a closed-mouth position, as it shows an area below the receiver operating characteristic curve of 0.671 (*p*-value = 0.028) and an accuracy of 71.4 using MR imaging as gold-standard [14].

In the studies mentioned above, the researchers took three measurements to improve the statistical power of their ultrasonographic measurements. However, in the daily clinical setting, i.e., outside this field of research, this procedure is a waste of time and resources in the diagnosis of TMD. Because clinical resources are limited, it is advisable to ensure the optimization of effort, energy, and economic cost. The main purpose of the study was to prove the reliability and validity of a single LCCD measurement instead of a 3-measurement approach to optimize time, economic cost, and effort in a clinical setting. The STARD guidelines for diagnostic accuracy studies have been followed [17].

## Material and methods

### Study design

Following the solution $n= \frac{2{({Z}_{\alpha }+ {Z}_{\beta })}^{2}*{S}^{2}}{{d}^{2}}$; where ${Z}_{\alpha }$ = Z risk α-value, ${Z}_{\beta }$ = Z risk β-value, ${S}^{2}$ is the variance of the differences, and $d$ = minimum value to be detected, we assume a 95% confidence level, power of 90%, an accuracy of 0.5 mm in the measurement, and a standard deviation of 0.45 mm [15], resulting in a sample size equal to 17 temporomandibular joints (TMJ), *n* = 20 adjusting for a 15% loss. A non-probability sampling method was used since the recruitment of volunteers took place at the Faculty of Health Sciences over a three-month period. The number of volunteers exceeded the required sample size. The study design was based on a test–retest analysis, where the first measurement of the LCCD was compared to the mean of three measurements taken later by the same examiner.

### Participants

Thirty-six adult participants, 12 men and 24 women aged between 19 and 54 years old, students and employees from the Camilo José Cela University (Madrid, Spain), were recruited. Participants were excluded if they presented neurological diseases; rheumatic diseases (rheumatoid arthritis, ankylosing spondylitis, etc.). They were also excluded if they had received physiotherapy treatment 15 days before their participation; prescribed pharmacological treatment with either analgesic, non-steroidal anti-inflammatory drugs, muscle relaxants (or any combinations of the above), for 48–72 h prior to the start of the study. Those participants that reported orofacial pain unrelated to TMD or any other underlying medical condition such as fracture, tumor, or trauma were also excluded.

The study was approved by the Institutional Ethics Committee of Camilo José Cela University (code number 09-22-UEMDTT) in accordance with the guidelines for the Helsinki Declaration. All sample subjects read and signed the written informed consent before participating in the study.

### Test methods

**Fig. 1:**
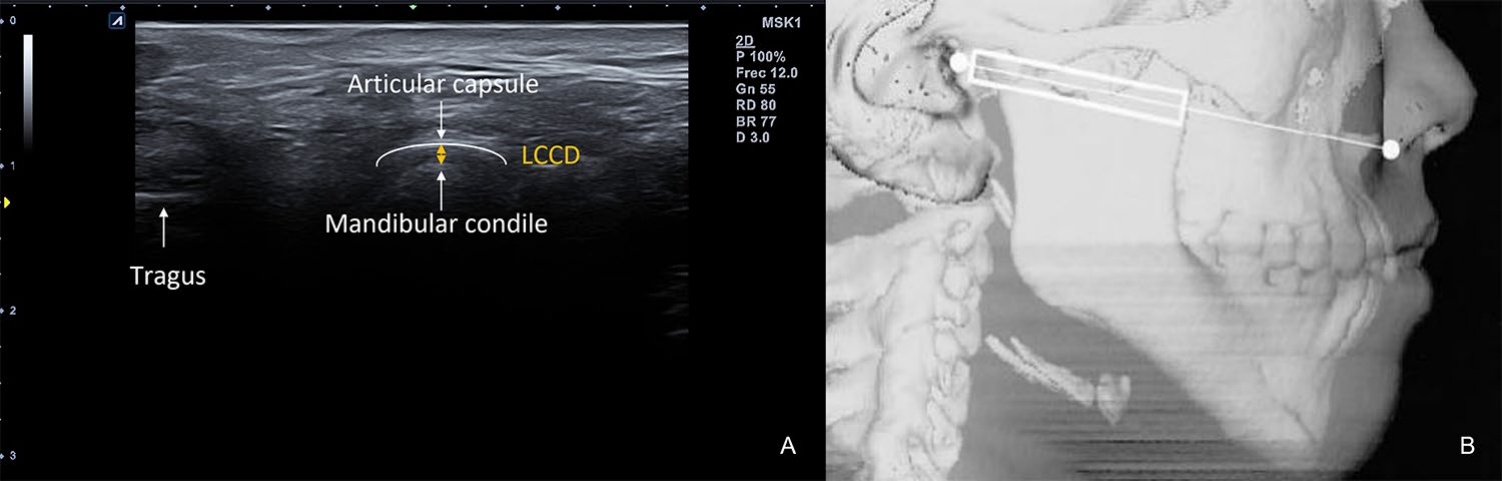
**A** US image obtained from the axial view. *LCCD* Lateral condyle-capsule distance. **B** US probe position schema. In: [16]. With the kind permission of the American Society of Neuroradiology

One examiner evaluated 72 TMJ of 36 Caucasian subjects who met the inclusion criteria. TMJ were assessed in a sitting position, in a closed mouth position and a neutral head and neck position. The examiner positioned the probe on an axial plane, running parallel to the Camper line (the line intersecting the ala of the nose and de tragus of the ear), perpendicular to the zygomatic arch, and parallel to the mandible ramus (Fig. 1). The probe was tilted to get the best view of the mandibular condyle and the TMJ. The dynamic movement of the TMJ was assessed in axial and coronal planes in closed-mouth and maximal open-mouth positions. Once the best image in the axial plane was obtained on the screen, it was recorded. All images were acquired with an Alpinion eCube i8 (Anyang-si, Gyeonggi-do, Ltd., Korea) with a 4 cm width linear transducer E8-PB-L3-12 T (frequency bandwidth 3–12 MHz) In the first round, the observer took one single LCCD measurement of the right and left joints as described by authors [14–16]. In a second round, one week later, the examiner evaluated the same 32 TMJ, but took three measurements of the LCCD, ensuring equal examination conditions. Each time he recorded the distance, he separated the probe from the subject's skin and restarted the process. For the study, researchers calculated the mean of these 3 measurements and compared them with the single measurement taken in the first round.

### Data analysis

#### Reliability

Intraobserver reliability was calculated using a two-way mixed effects model, absolute agreement type, and single measure of intraclass correlation coefficients (ICC) (2,1), as proposed by Shrout and Fleiss [18]. This method allows for the confidence and reliability of the single observer to measure the same anatomical landmark. ICC values were considered using the Portney and Watkins classification, where a 0.50–0.75 value was judged moderate, a value ≥ 0.75 was classified as good, and ≥ 0.90 was considered excellent [19].

#### Validity

A Bland–Altman plot was designed to analyze the agreement between the two measurements [20]. This method permits a graphic form to evaluate a measurement against its gold standard, observing how much the differences deviate from the average when comparing different measurements. In this case, no gold standard exists, but we show how far the single measurement in the TMJ is from the 3-measurement average. These authors contemplated a Limits of Agreement (LoA) as the mean difference ± 1.96 standard deviations of the difference, which would include 95% of the measurements taken with the analyzed method. The Bland–Altman plot calculates the bias between methods and the degree of variability through the scatter of measures with respect to the bias line.

The significance level was set at *p* ≤ 0.05. All statistical analyses were performed using the SPSS package (IBM SPSS Statistics for Macintosh, Version 25.0. Armonk, NY, US: IBM Corp.)

## Results

Means and standard deviations were calculated for all the measures carried out. The single-measure showed 1.55 ± 0.42 mm, whereas measures 1 and 3 showed 1.57 ± 0.42, and measure 2 was 1.56 ± 0.41 mm. The 3-measurements average was 1.57 ± 0.41, with a standard error equal to 0.049. The difference between the single-measure and the 3-measurements average was 0.019 ± 0.08 mm. The descriptive data of analysis are resumed in Table 1.

**Table 1:** Descriptive data

	Mean ± SD	Min–Max	SE
Measure 1	1.57 ± 0.42	0.90–3	0.050
Measure 2	1.56 ± 0.41	0.90–3	0.048
Measure 3	1.57 ± 0.42	0.90–3.10	0.049
Single-measure	1.55 ± 0.42	0.90–3	0.049
Difference	0.019 ± 0.08	− 0.20–0.17	0.009
Age	34.22 ± 9.24	19–54	1.52

*SD* standard deviation, *Min*–*Max* minimum–maximum values, *SE* standard error

**Fig. 2:**
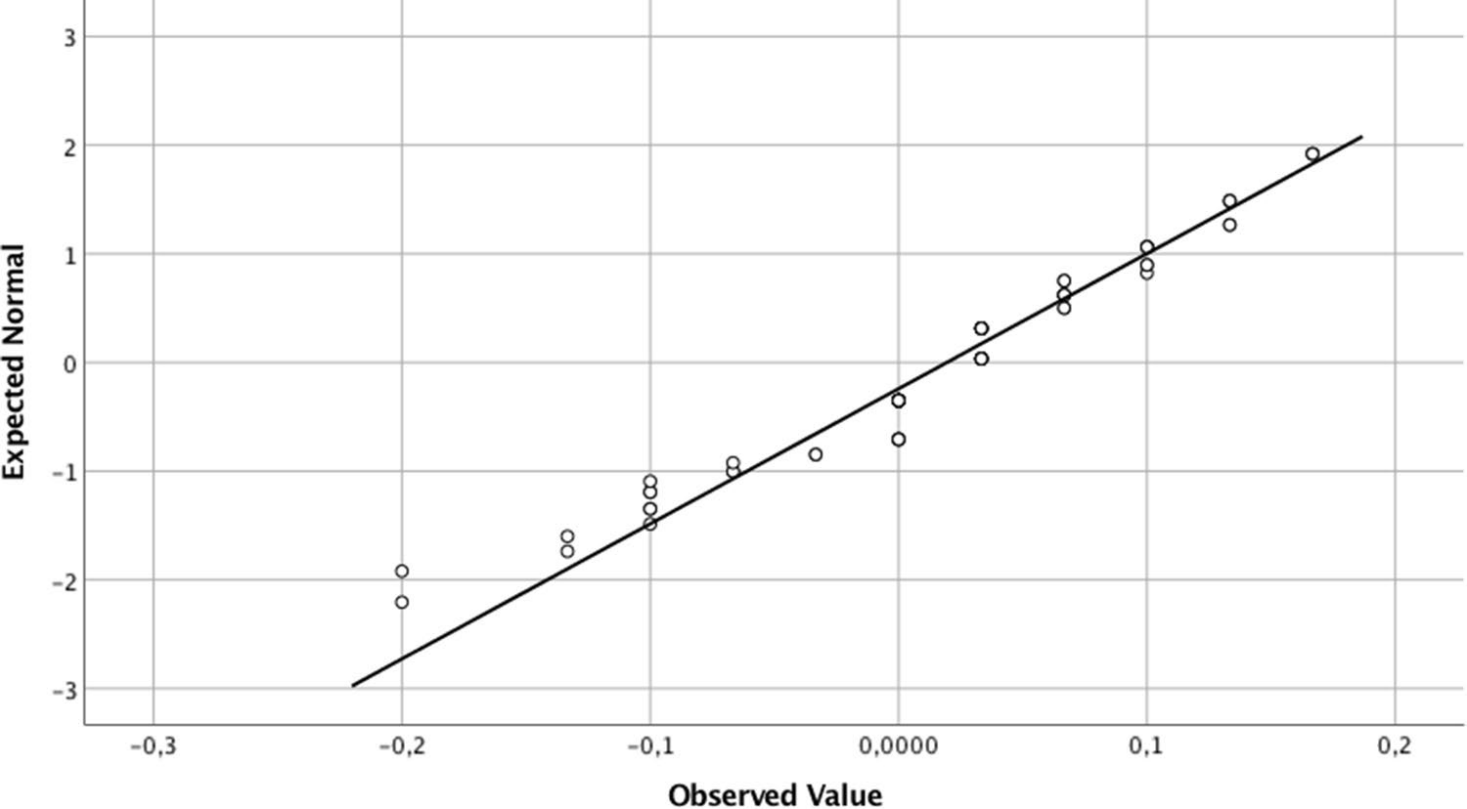
Normal quantile-quantile plot for the differences between measurements (mm). It shows a small deviation from the expected normal values, allowing to assume a normal distribution of the data

The Kolmogorov–Smirnov test revealed a non-normal distribution of the differences (*p* < 0.001), but the Q-Q plot and the histogram showed a moderate kurtosis and skewness that allowed the assumption of normal distribution (Fig. 2).

Therefore, a two-way mixed model ICC_(2,1)_ and Bland–Altman plot were used to study the intraobserver reliability and the validity of the single measurement against the 3-measurement average.

### Reliability

The calculated ICC_(2,1)_ between the single-measure and the 3-measurements average shows intraobserver reliability of 0.981, and 95% confidence intervals (CI) of 0.969 to 0.988, in keeping with Portney and Watkins [19], authors who rate that value as excellent.

### Validity

**Fig. 3:**
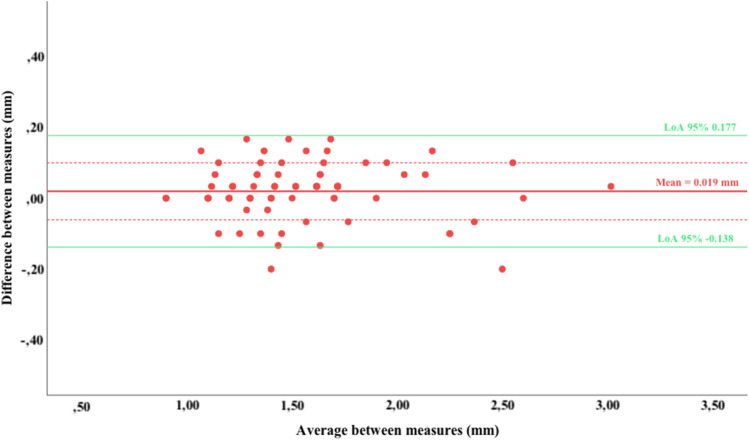
Bland–Altman plot of the mean difference and 95% limits of agreement for the US measurements

There is a mean difference between the sonographic measurements of 0.019 mm (95% CI from 0.0005–0.0383), with a standard deviation of 0.080 mm (Fig. 3). The 95% LoA, calculated as bias ± (1.96 * standard deviation of the difference), were − 0.138 for the lower and 0.177 for the upper limit. The mean relative error was 0.009 mm.

## Discussion

This study was designed to test the reliability and validity of a single LCCD measurement against a 3-measurement approach to save time, economic cost, and effort in a clinical setting. Our results indicate a high level of coincidence between one single measurement and the calculated mean of three measurements.

### Clinical relevance

The use of US for TMD is being studied widely. There is a clear disagreement among authors on how US can contribute to disc visualization and this disagreement is clearly explained in corresponding studies. Some authors argue that US technology can visualize the condition and position of the disc [21–26], while others describe the difficulty of US to clearly see the disc and determine its location, using other items to estimate TMD [14–16, 27].

In the authors’ opinion, disc visualization with US is poor. Only a small part can be observed and only in some patients. So, it may be more effective to focus the US examination on indirect signs of disc damage or disc displacement, as the LCCD measurement does. In this sense, the review of Kundu et al. concludes that US offers an acceptable level of sensitivity in the diagnosis of disc derangement [27], similar to Talmaceanu et al. [28] who report a sensitivity of 93.1%, a specificity of 87.88%, and accuracy of 90.32% compared to MRI as gold-standard when assessing the joint disc displacement. In a similar study, Jank et al. [29] found 92% sensitivity, specificity, and accuracy comparing disc displacement between MRI and high-resolution US in 200 temporomandibular joints in a closed-mouth position. However, certain authors emphasize the greater specificity of US for sensitivity in assessing TMJ disc location, which impacts the positive and negative predictive values of the diagnosis, and they criticize the LCCD as a landmark of anterolateral displacement because it requires anatomical changes, like joint capsule enlargement or joint effusion [25].

In our opinion, dynamic US exploration is what adds value to the TMJ assessment, not only because of the scope of measurements to be taken, but also because of the movement we can see in the dynamic exploration, assessing condylar erosions, articular effusion, and even feeling the “click” of the disc displacement while we explore in a closed and open-mouth position.

### Measurement accuracy

The bias between US methods is 0.019 mm, as shown in the Bland–Altman plot. Despite being statistically significant, it does not seem to have an impact on the diagnosis of pathology associated with disc joint displacement, since authors determine greater LCCD measurements when assessing TMD. Hence, the presence of joint effusion is considered when LCCD is more than 2 or 3 mm [27]. Moreover, the sensitivity and specificity of US versus MRI vary significantly for each 0.10 mm cut-off value [30]. These researchers point out the importance of reliable interobserver data since a difference of as little as 0.2 mm in the capsular distension measurement could impact on either true or false positive rates. As stated earlier, Hayashi et al. comment on internal joint derangement when the LCCD is ≥ 3–4 mm (depending on the population studied), but they do not observe a cut-off value below 1 mm, probably due to the technology used at the time [16].

More current values of Çakır-Özkan et al. [14] equally show a cut-off value of 1 mm. Even considering the reported minimum cut-off value, the limits of agreement of this study are below 0.2 mm in absolute values, indicating that future measurements using this single sonographic method will fall within − 0.138 and 0.177 mm in the 3-measurements method.

The US equipment used in this study is like any equipment used for musculoskeletal (MSK) imaging in clinical settings. The examiner had no previous experience in TMJ US, but had more than ten years of experience in MSK US. The examiner carried out a pilot study with 20 TMJ as training for this study. It is important to understand TMJ anatomy and US technique for this exploration, but after this knowledge is acquired, the exploration technique is not difficult.

### Intraobserver reliability

The LCCD measurement has good intra-observer reliability, as Elias et al. show in a study of 30 TMJ [15]. Their ICC values were 0.83 both for longitudinal and transversal scans in the closed-mouth position. In our study, the ICC values were higher, perhaps due to an improvement in device technology. The review of Kundu et al. does not reflect statistical values regarding US measuring reliability, as it shows intra-observer and inter-observer percentages for the detection of the disc position, ranging between 87 and 93% for the former and between 82 and 90% for the latter [27]. Despite obtaining high values, we do not consider percentages a good indication of reliability in a study since it does not consider certain biases, such as agreement by chance.

### Limitations

The main limitation of this study is the use of a single observer, which does not allow for the analysis of inter-rater reliability or for the estimation of method validity from another observer. The second potential source of error relies on the absence of comparison between US measurements and any other technique, although TMJ measuring has been widely studied alongside MRI and TC imaging with reliable results [14, 16, 26, 31].

Moreover, during the period between measurements, some TMJ changes may have occurred, and the status of the joint at the time of the second US examination could have been slightly different from those visualized at the time of the first trial.

## Conclusions

The intra-observer reliability of a trained examiner is remarkably high in the measurement of the LCCD, conveniently respecting the anatomical landmarks. Moreover, the risk of bias is very low in the measurement when comparing the mean of three LCCD measurements as opposed to a single measurement, which makes the technique valid for use in a clinical setting, while saving on resources, economic cost, and effort.

## Data Availability

The sample data is available upon contact with the authors.
